# The “Omega sign”: a new radiological sign for a rare type of internal hernia involving the sigmoid mesocolon

**DOI:** 10.1259/bjrcr.20190127

**Published:** 2020-09-29

**Authors:** Kino Ceon Francis, Candice Daley, Bonnie-Paul Regis Williams, Richard Bullock, Ulanda Singh, Akil Baker

**Affiliations:** 1 Department of Diagnostic and Interventional Radiology, University Hospital of the West Indies, Mona, Jamaica; 2 Department of Surgery, University Hospital of the West Indies, Mona, Jamaica

## Abstract

The transmesosigmoid hernia is a rare type of sigmoid mesocolon hernia. Its presentation is non-specific and thus hardly ever preoperatively diagnosed. Its diagnosis often requires surgical corroboration. This case report aims to improve on the preoperative diagnosis with a proposed observed sign on CT. All literature reviewed described radiological findings related to the small bowel; thus, features of small bowel obstruction was the “hallmark” of internal hernias. This paper intends to describe the features of the sigmoid mesocolon internal hernias, illustrate and propose a never reported configuration of the sigmoid colon. This sigmoid colon configuration has a resemblance to the omega sign. We intend to present a new hallmark sign, which may serve as a clue in the identification of internal hernias involving the sigmoid mesocolon.

## Case presentation

A 65-year-old female presented to our institution with recurrent lower abdominal pain for greater than 1 month. She was known to have diabetes mellitus type 2, hypertension and gave a history of total abdominal hysterectomy 1 year prior for abnormal uterine bleeding. Her symptoms were previously thought to be due to a urinary tract infection, and she was treated for the same; however, her symptoms did not resolve. She presented with acute worsening of the lower abdominal pain 4–6 h before the presentation. The pain initially confined to the suprapubic region and then became generalized and constant. This pain worsened upon sitting up or extending the legs. She also reported bilious vomiting but had no history of fever, constipation or obstipation. She reported constipation, which began 6 months before presentation with a change in calibre to small pellets along with night sweats and weight loss. No history of bleeding per rectum reported. She had no prior screening colonoscopy. On examination, her vital signs were all normal, and significant findings confined to the abdominal examination. She was obese, with a surgical midline scar and a small umbilical hernia noted. Tenderness and voluntary guarding elicited in the entire lower abdomen. Bowel sounds and rectal examination were normal.

## Investigation

**Figure 1.**
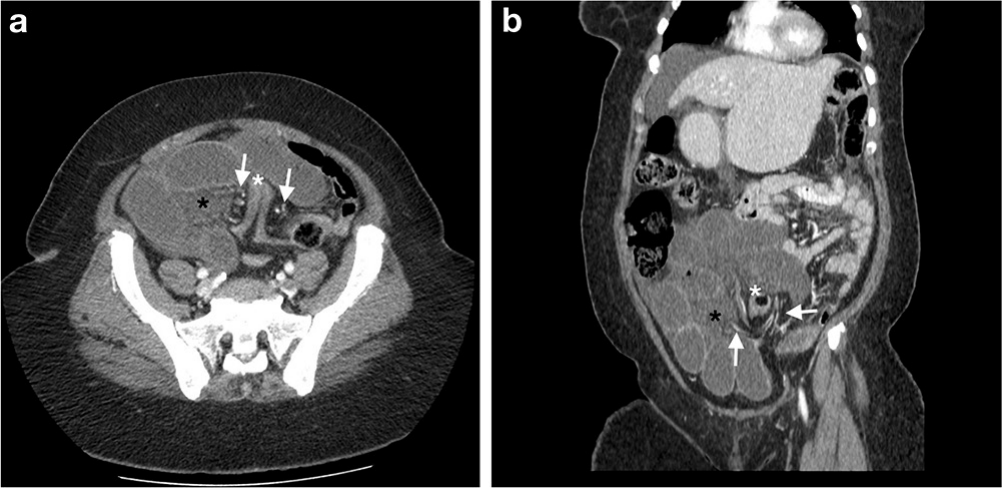
Axial and coronal images demonstrated stretching of the mesenteric vessels (left short arrow) on the left passing beneath the tethered sigmoid colon, through both peritoneal layers lead by loops of dilated fluid-filled ileum resulting in a closed-loop obstruction. These loops of ileum and mesenteric vessels (right short arrow) exited on the right. The white asterisk shows a tented sigmoid colon forming an apex with collapsed sigmoid colon on either side. The black asterisk indicates the associated steamy mesentery appearance, a feature of small bowel ischaemia. The cluster of dilated fluid-filled loops of ileum also demonstrated poor enhancement compared to the loops of jejunum in the left upper quadrant consistent with ischaemia (b). The mesenteric vessels were, however, of normal contrast media opacification. Moderate intra-abdominal-free fluid observed in the right subphrenic, perihepatic, right para-colic and pelvic regions.^1^

**Figure 2.**
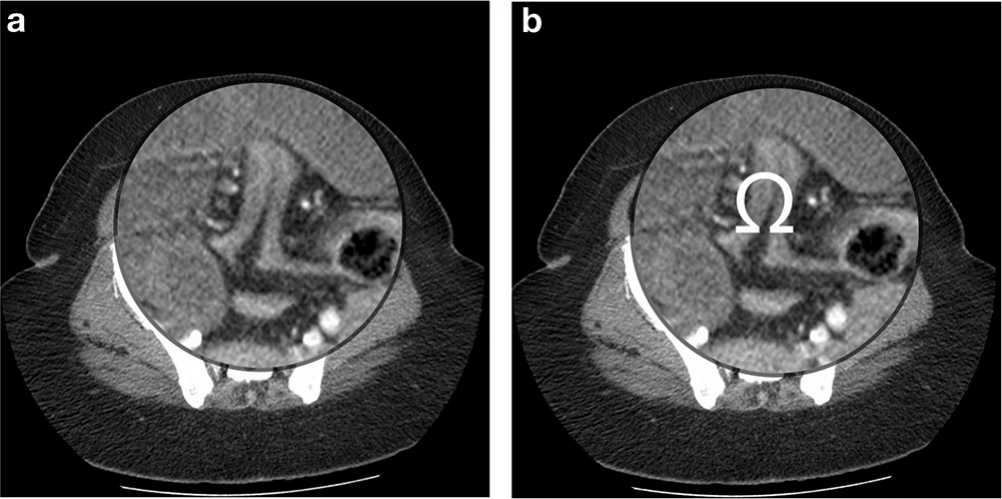
Axial images demonstrated a characteristic appearance of a tented collapsed sigmoid colon under which the ileum and mesentric vessels herniated. The appearance is that of an “Omega sign” which we are ascribing and proposing to be a useful clue to the underline process of this type of closed-loop obstruction.

Significant biochemical and haematological findings were lactate of 6.0 mmol l^−1^ (N 0.5–1.0) and white blood cell count (WBC) of 15.0 × 10^9^/L (N 4.0–11.0). All other parameters were normal. A CT scan of the abdomen and pelvis was done in the portovenous phase (at 65 sec.) with 1.25 mm axial, sagittal and coronal reconstructions. The CT scan showed high-grade bowel obstruction with hypoattenuating bowel wall, consistent with ischaemia. The detailed findings are demonstrated in the figures below (Figures 1 and 2).

## Treatment

**Figure 3.**
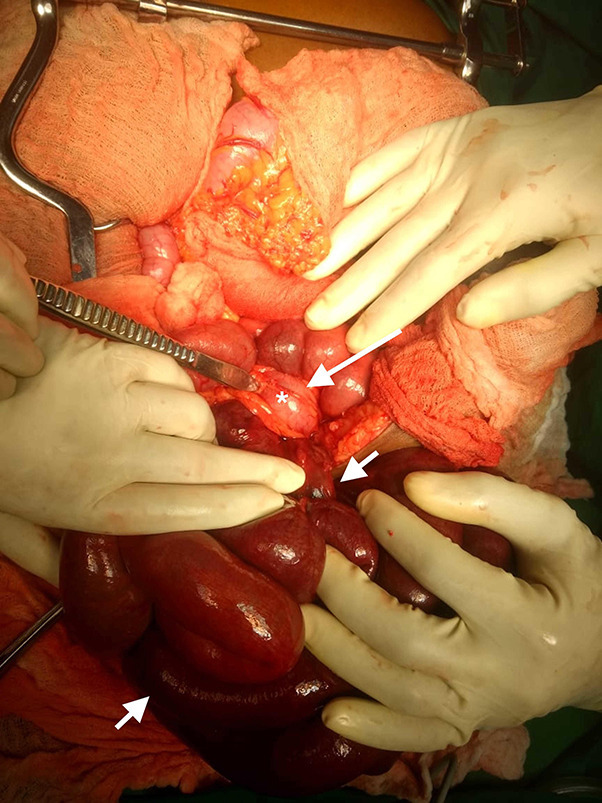
Intraoperative photograph demonstrated the segment of sigmoid colon and mesentery under which the ileum herniated through. Short arrows indicate the 260.0 cm of gangrenous ileum without a hernia sac. Proximal resection point was 330.0 cm from the duodenojejunal flexure while the distal resection point was 10.0 cm proximal to the ileocaecal valve. The long arrow shows the tented collapsed sigmoid colon (white asterisk) at the apex.

The patient had exploratory laparotomy, which revealed gangrenous ileum, approximately 260.0 cm in length. This gangrenous segment began 330.0 cm from the duodenojejunal flexure. It ended 10.0 cm proximal to the ileocecal valve. This segment of gangrenous bowel was noted to be herniating through a defect in the sigmoid mesocolon, which caused a closed-loop obstruction. A significant potential pitfall noted intraoperatively was that a tented collapsed sigmoid colon (white asterisk) was present. Because of this appearance, it was initially thought to be a thick adhesive band as the gangrenous herniating ileal segments traversed beneath it. Fortunately, the sigmoid colon was not transected, and after further inspection, the defect on the mesocolon identified. An adhesive band tethering the proximal limb of the sigmoid loop to the pelvic sidewall was found. This adhesive band was of predominantly fatty tissue and was adherent from the left leaflet of the sigmoid mesocolon to the pelvic sidewall. The defect in the mesocolon might have been iatrogenic from the patient’s first surgery. After the adhesive band was lysed, the ileum was reduced and resected, and an ileo-ileal anastomosis was done. The sigmoid colon returned to its normal anatomic position, and the mesocolon closed (Figure 3).

## Outcome and follow-up

The postoperative course was uneventful. The patient was discharged with plans for followed-up in the surgical outpatient department.

## Discussion

There are two main categories of bowel-related abdominal hernias, external hernias and the internal hernias. This case looks to examine the rarer internal hernia, which is a protrusion of a viscus through a foramen or fossa within the abdominal cavity.^1^ Internal hernias often present as small bowel obstruction and represent 0.5 to 4.1% of all cases.^2^ Internal hernias can be further classified according to their location. The hernia of interest belongs to the rare group of internal hernias that Benson and Killen et al identified as sigmoid mesocolon hernias (intersigmoid, transmesosigmoid and intramesosigmoid).^3^ The transmesosigmoid hernia is incarceration of loops of bowel through an isolated, oval defect in the left and right leaves of the sigmoid mesocolon. This hernia is distinguishable from the intramesosigmoid hernia as no hernia sac is present since it involves both of the peritoneal layers. The intramesosigmoid hernia on the other hand involves the right or the left leaf of the sigmoid mesocolon. This is associated with a hernia sac since the abnormal defect involves only one peritoneal layer.^3,4^ The transmesosigmoid hernia is thought to progress rapidly with a higher incidence of strangulation due to the lack of a hernial sac.^5^ The intersigmoid hernia subtype involves the incarceration of bowel loops through a congenital fossa located at the attachment of the lateral aspect of the sigmoid mesocolon with the obstructed small intestine behind the mesentery of the sigmoid colon.^6^ Sigmoid mesocolon hernias represent approximately 5.0 to 6.0% of all internal hernias. Benson and Killen et al described only 34 cases reported at the time of their investigation with a moderate increase since then but remains a rare condition. Kayano et al reported that the intrasigmoid subtype accounted for approximately 50.0–57.3% of sigmoid mesocolon hernias while the intersigmoid and transmesosigmoid hernias are responsible for 24.5–35.0% and 15.0–18.0%, respectively, in Japan, a total of approximately 64 reported cases.^6^ Benson and Killen et al shared a different experience where they found in their report of 34 cases that the intrasigmoid subtype accounted for only 3.0% of sigmoid mesocolon hernias, intersigmoid for the majority of 88.2% and transmesosigmoid hernias being responsible for 8.8% of their cases.^3^ The Japanese literature shows a male predominance for intersigmoid and intramesosigmoid hernias while for transmesosigmoid hernias, there is no sex predilection.^7^ This, however, deviates from that reported by Bin Li et al who found from a review of 27 cases that transmesosigmoid hernias are slightly more common in males than females with a ratio of 1.44:1, and a mean age of 53.1 years.^5^ These were the largest documented case series found in the literature. Our patient was female of 65.0 years of age; with a presentation of progressive and persistent symptoms of intestinal obstruction along with a history of previous surgery. An incarcerated hernia (internal or external) should be in the differential list.^5,8^ Other considerations would include bands and adhesions, a non-benign lesion as well as a volvulus. CT is the gold standard imaging technique as it is quick, readily available, and provides much detail. With the advent of multidector CT (MDCT), which produces thin-section axial images and high-quality multiplanar reformations (MPRs), there has been an improvement in the visualization of normal anatomy and pathology. This has lead to an improvement in the preoperative diagnosis of internal hernias as a cause of small bowel obstruction.^4^

It has remained a challenge for the radiologist to diagnose internal hernias of the sigmoid mesocolon on CT and also to further distinguish between the different subtypes preoperatively. Knowledge of the anatomy of the peritoneum is always helpful. Previous literature mainly spoke to the evidence of small bowel obstruction and described a sac-like mass, C-shaped dilated loops of small bowel with collapsed distal segments which suggest the possibility of an internal hernia. In several instances the impression was small bowel obstruction of unknown aetiology with no consideration of an internal hernia of the sigmoid mesocolon as a distant differential diagnosis. It has been reported that the findings on CT for transmesosigmoid hernia is similar to that of intrasigmoid hernia making more specific CT diagnosis even more challenging.^6,8^ There is the general opinion in the literature that hernias of the sigmoid mesocolon are featureless and imaging tests are non-specific.^5^ One feature that helps the radiologist to make a distinction is the displacement of the anteromedial sigmoid colon due to entrapped bowel loops behind the left posterior or lateral aspect of the sigmoid colon. These findings would be more in keeping with a transmesosigmoid hernia.^5^ Surgery is the mainstay of diagnosing these hernias. Transmesosigmoid hernia is usually incarcerated with gangrenous bowel owing to the absence of a hernia sac. The size of the defect is also related to the likelihood of incarceration, the average size causing incarceration being 3.4 cm with a range of 2.0–5.0 cm and the average length of ileum resected is 44.5 cm.^5^

Our female patient was found to have a transmesosigmoid hernia with an anomaly of an adhesive band of fatty tissue arising from the left leaflet of the sigmoid mesocolon resulting in the collapsed sigmoid colon assuming an “Omega sign” appearance on the CT axial images. Through both layers of the sigmoid mesocolon herniated 260.0 cm of ischaemic ileum which was resected. These findings represent a variant of the transmesosigmoid hernia subtype. In our literature review, we did not encounter such a description of the appearance of the sigmoid colon or the association with an adhesive band of fatty tissue. We, therefore, suggest that this appearance and description of the sigmoid colon be incorporated as a possible clue to the cause of closed-loop small bowel obstruction in the region of the sigmoid colon. If seen, the radiologist should give more consideration for a sigmoid mesocolon internal hernia as the underlying cause of the small bowel obstruction, (likely of the transmesosigmoid subtype) especially in the absence of a hernia sac. The finding of the adhesive band of fatty tissue may or may not be related to the patients’ previous pelvic surgery. Reports of adhesions (not fatty tissue) attached to the herniated ileum in patients with no previous abdominal surgery have been documented.^9^ We did not encounter reports of adhesions attached to the sigmoid colon in cases of sigmoid mesocolon internal hernias. The nature of the adhesive band of fatty tissue found intraoperatively in this case is therefore uncertain.

We propose a new sign the “Omega sign” as a possible added clue to the preoperative CT diagnosis of sigmoid mesocolon hernias and more specifically the transmesosigmoid subtype. The sensitivity and specificity of the “Omega sign” are yet to be determined as only a few score of cases of sigmoid mesocolon internal hernias have been reported in the literature to date, with only a few of these being the transmesosigmoid subtype.

## Learning points

- Internal hernias are rare with the sigmoid mesocolon type being even more rarer than other types.
- These hernias frequently present with small bowel obstruction along with ischaemic bowel segments.
- While plain abdominal radiographs are the initial radiological investigation in a patient with symptoms of intestinal obstruction, a CT scan of the abdomen and pelvis is the gold standard for identifying the cause of intestinal obstruction, being able to highlight key features of the underline cause.
- Adhesions can complicate underlying internal hernias, resulting in further complex radiological and surgical appearance.
- The “Omega sign” seen on the CT axial images of the abdominopelvis may be a useful sign in the preoperative CT diagnosis and identification of sigmoid mesocolon hernias.
- Without a heightened awareness along with a good appreciation for the anatomical and pathological dynamics of internal hernias of the sigmoid mesocolon, there will be frequent misdiagnosis or delayed diagnosis resulting in significant morbidity and mortality.
